# Supplementary material for: Breakdown of energy transfer gap laws revealed by full-dimensional quantum scattering between HF molecules
Source: Nat Commun. 2019 Oct 11;10:4658. doi: 10.1038/s41467-019-12691-8 (PMC6789015; doi:10.1038/s41467-019-12691-8)
Supplement: Supplementary file 2 — Supplementary Information [file 41467_2019_12691_MOESM2_ESM.pdf]

Breakdown of Energy Transfer Gap Laws Revealed by Full-Dimensional Quantum Scattering between HF Molecules

Yang et al

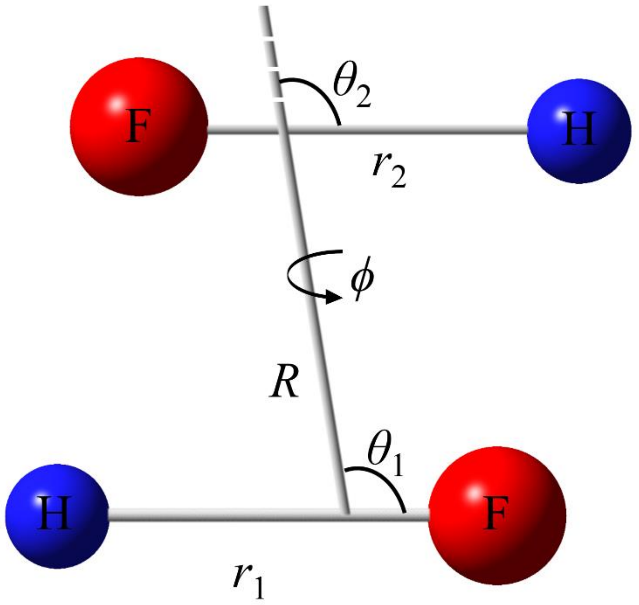

Supplementary Fig. 1 Body-fixed Jacobi coordinates (R, r_1, r_2, θ_1, θ_2, ϕ) for the HF–HF system.

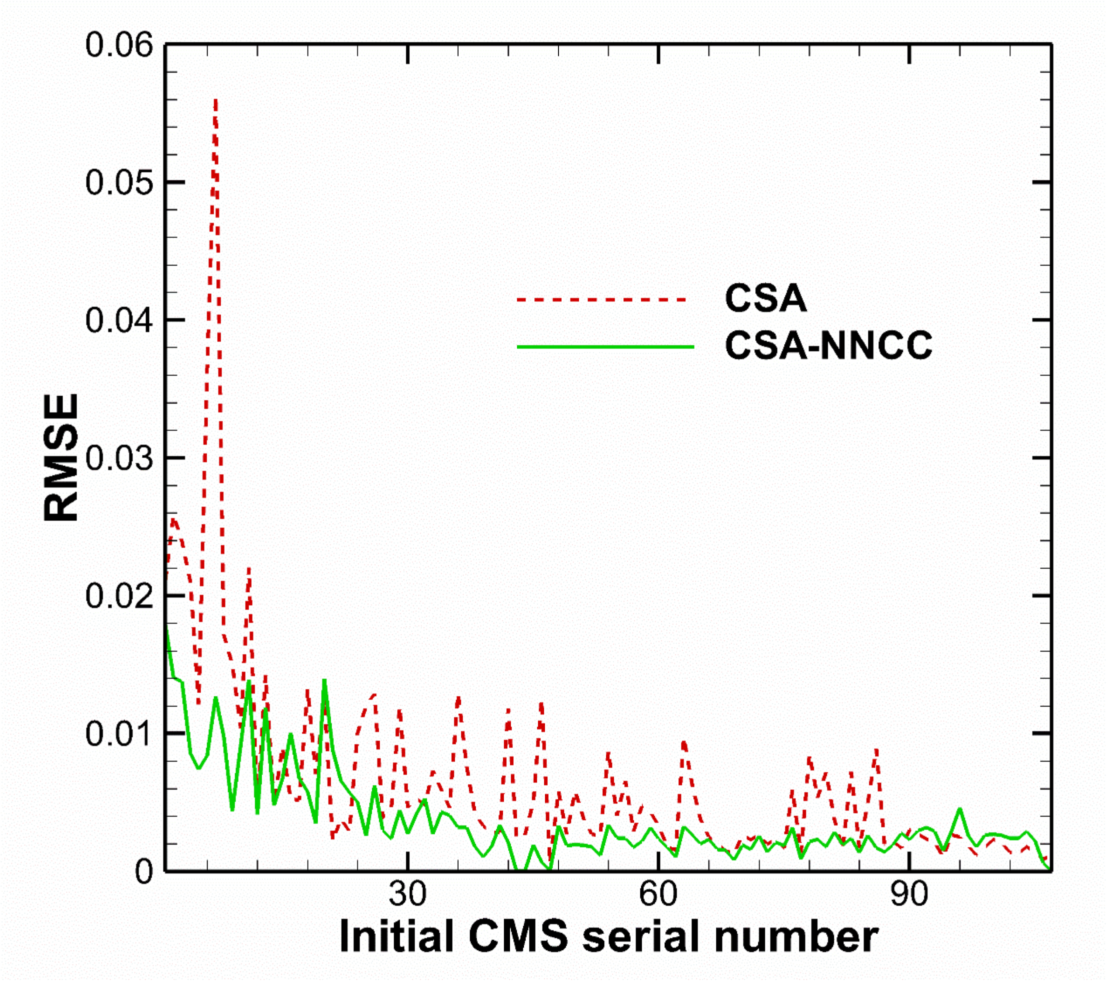

Supplementary Fig. 2 RMSEs of the state-to-state probabilities from various initial CMSs. Only probabilities obtained by using rigorous CC approach that are larger than 0.01 are included. The total energy is fixed at E=1.133 eV and the total angular momentum and the parity are chosen as $J(P_{ex}p\varepsilon)$=10(+−+). There are 107 initial CMSs at this total energy and they are arranged in the sequence of the sum of two diatomic rotational quantum numbers j_1+j_2 from a high value to a low one.

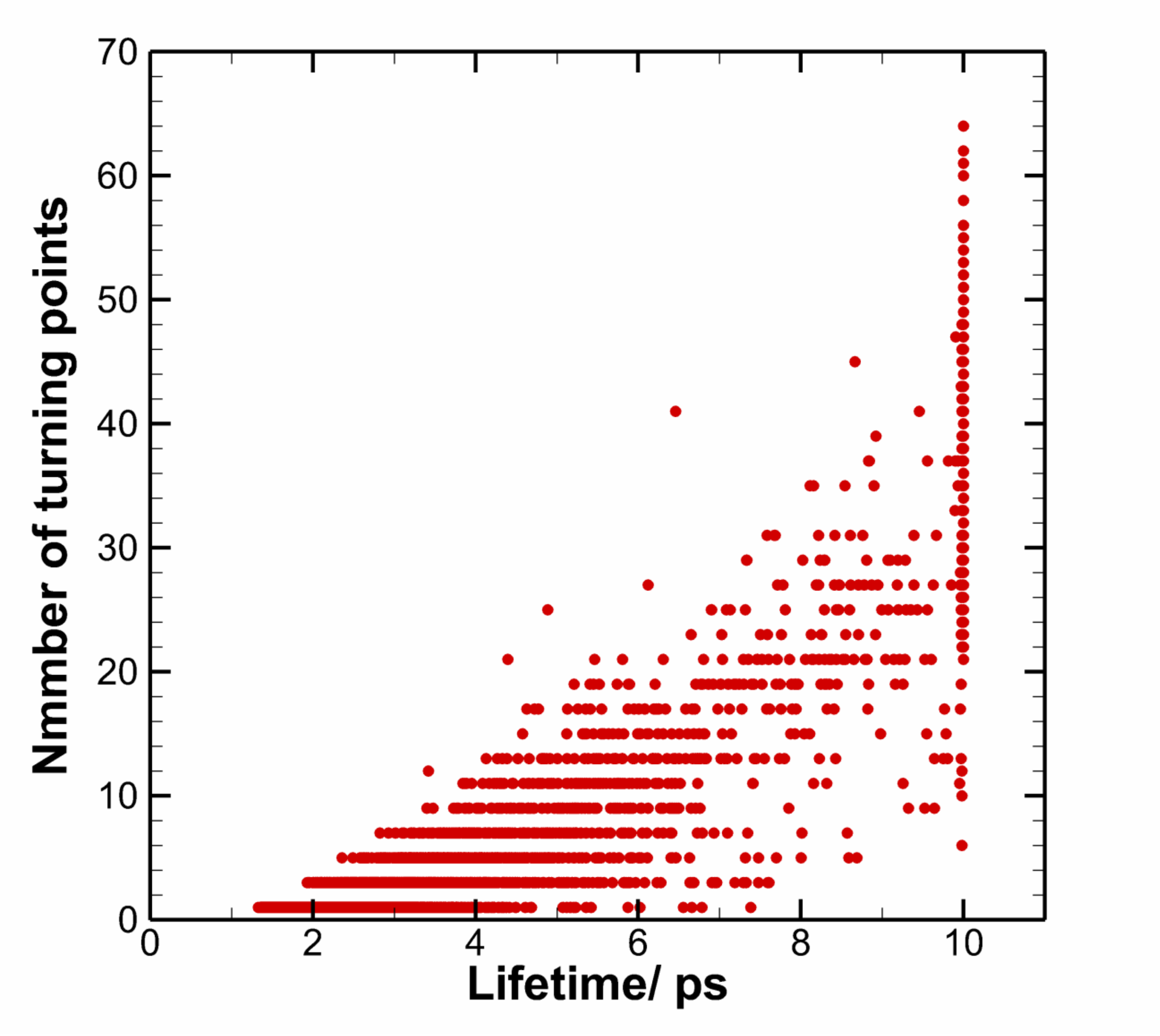

Supplementary Fig. 3 The lifetime distributions for trajectories with different numbers of inner turning points. The last column include all trapped trajectories at the end of the 10 ps propagation.

Supplementary Note 1

For diatom–diatom scattering problems, it is convenient to use the diatom-diatom Jacobi coordinate system (R, r_1, r_2, θ_1, θ_2, ϕ), as shown in Supplementary Figure 1. The Hamiltonian in this coordinate system has the following form,

$$H = -\frac{\hbar^2}{2\mu}\frac{\partial^2}{\partial R^2} + \frac{\left(\mathbf{J}-\mathbf{j}_{12}\right)^2}{2\mu R^2} + h_1\left(r_1\right) + h_2\left(r_2\right) + \Delta V\left(R, r_1, r_2, \theta_1, \theta_2, \phi\right), \tag{1}$$

where μ is the reduced mass of the HF–HF system and ΔV is the interaction potential between two diatoms. $h_i\left(r_i\right)$ are diatomic ro-vibrational Hamiltonians expressed as

$$h_i\left(r_i\right) = -\frac{\hbar^2}{2\mu_i}\frac{\partial^2}{\partial r_i^2} + \frac{\mathbf{j}_i^2}{2\mu_i r_i^2} + V_i\left(r_i\right), \tag{2}$$

where μ_i are the reduced masses of the diatoms. The corresponding Schrödinger equations are $h_i\left|v_i j_i\right\rangle = E_{v_i j_i}\left|v_i j_i\right\rangle \left(i=1,2\right)$, in which $\left|v_i j_i\right\rangle$ are the ro-vibrational eigenfunctions labeled by the vibrational and rotational quantum numbers v_i and j_i, respectively, and $E_{v_i j_i}$ are their energy eigenvalues.

The total scattering wave function can be expanded as

$$\left|\Psi^{\pm Jp\varepsilon}\right\rangle = \sum_{vjK} F_{vjK}^{\pm Jp\varepsilon}\left(R\right)\left|X_{vjK}^{\pm Jp\varepsilon}\right\rangle, \tag{3}$$

where the symmetrized body-fixed (BF) ro-vibrational functions serve as the basis for the system including two identical molecules,[1]

$$\left|X_{vjK}^{\pm Jp\varepsilon}\right\rangle = \Delta_{vj,\tilde{v}\tilde{j}}\left[\left|Y_{jK}^{Jp\varepsilon}\right\rangle\left|\Phi_{vj}\right\rangle \pm \varepsilon\left(-1\right)^{j_{12}}\left|Y_{\tilde{j}K}^{Jp\varepsilon}\right\rangle\left|\Phi_{\tilde{v}\tilde{j}}\right\rangle\right]. \tag{4}$$

The normalization factor $\Delta_{vj,\tilde{v}\tilde{j}} = \left[2\left(1+\delta_{v\tilde{v}}\delta_{j\tilde{j}}\right)\right]^{-1/2} = \left[2\left(1+\delta_{v_1 v_2}\delta_{j_1 j_2}\right)\right]^{-1/2}$. The radial basis is a direct product of two diatomic ro-vibrational eigenfunctions,

$$\left|\Phi_{vj}\right\rangle = \left|v_1 j_1\right\rangle \left|v_2 j_2\right\rangle, \tag{5}$$

and $\left|Y_{jK}^{Jp\varepsilon}\right\rangle$ are parity-adapted BF total angular momentum eigenfunctions in the coupled angular momentum representation[2]

$$\begin{aligned}\left|Y_{jK}^{Jp\varepsilon}\right\rangle = \frac{1}{\sqrt{2+2\delta_{K0}}}\Big[\left|JMK\right\rangle \left| j_1 j_2 j_{12} K\right\rangle \\ +\varepsilon(-1)^{J+j_1+j_2+j_{12}}\left|JM(-K)\right\rangle \left| j_1 j_2 j_{12}(-K)\right\rangle\Big]\end{aligned}. \tag{6}$$

In Supplementary equation (6), $\left|JMK\right\rangle$ is the normalized Wigner rotation matrix defined in terms of the Euler angles[3]

$$\left|JMK\right\rangle = \sqrt{\frac{2J+1}{8\pi^2}} D_{K,M}^{J*}(\alpha,\beta,\gamma), \tag{7}$$

where M and K are the projections of total angular momentum J onto the space-fixed (SF) and BF z axis, respectively[4]. And $\left| j_1 j_2 j_{12} K\right\rangle$ is expressed as

$$\left| j_1 j_2 j_{12} K\right\rangle = \sum_m \left\langle j_1 m j_2 (K-m) \middle| j_{12} K\right\rangle Y_{j_1}^m(\theta_1,0) Y_{j_2}^{K-m}(\theta_2,\phi), \tag{8}$$

where $\langle\cdots|\cdots\rangle$ is the Clebsch-Gordan coefficient and $Y_j^m(\theta,\phi)$ is the spherical harmonic function[3]. In this representation, $\mathbf{j}_1$ and $\mathbf{j}_2$ are coupled to $\mathbf{j}_{12}$, which is subsequently coupled with the orbital angular momentum $\mathbf{l}$ to yield the total angular momentum $\mathbf{J}$. For the total parity p=+1, the quantum number K ranges from 0 to J; otherwise (p=−1), K ranges from 1 to J. Note the restriction that p=+1 for J=0, and that for v_1=v_2 and j_1=j_2 case, the allowed j_{12} quantum numbers must satisfy the condition $P_{\mathrm{ex}}\varepsilon(-1)^{j_{12}} = 1$ [5]. We used the collective indices $v \equiv (v_1, v_2)$ and $j \equiv (j_1, j_2, j_{12})$, while $\tilde{v}$ and $\tilde{j}$ denote exchange of the corresponding indices respectively, i.e. $\tilde{v} \equiv (v_2, v_1)$ and $\tilde{j} \equiv (j_2, j_1, j_{12})$.

Substituting the Hamiltonian and the basis functions into the time-independent

Schrödinger equation leads to the close-coupling (CC) equations,

$$\left(\frac{d^2}{dR^2}+k^2_{v_1j_1v_2j_2}\right)F^{\pm Jp\varepsilon}_{vjK}=\sum_{v'j'K'}\left(2\mu\mathbf{V}^{\pm Jp\varepsilon}_{vjK,v'j'K'}+\frac{1}{R^2}\mathbf{U}^{\pm Jp\varepsilon}_{vjK,v'j'K'}\right)F^{\pm Jp\varepsilon}_{v'j'K'}, \tag{9}$$

in which $k_{v_1j_1v_2j_2}=\sqrt{2\mu\left(E-E_{v_1j_1}-E_{v_2j_2}\right)}$ is the channel wave number and E is the total energy of this system. The symmetrized interaction potential matrix $\mathbf{V}^{\pm Jp\varepsilon}$ and the centrifugal matrix $\mathbf{U}^{\pm Jp\varepsilon}$ can be written explicitly in terms of their corresponding unsymmetrized counterparts, respectively,

$$\mathbf{V}^{\pm Jp\varepsilon}_{vjK,v'j'K'}=2\Delta_{v_1j_1,v_2j_2}\Delta_{v'_1j'_1,v'_2j'_2}\left[\mathbf{V}^{Jp\varepsilon}_{vjK,v'j'K'}\pm\varepsilon(-1)^{j_{12}}\mathbf{V}^{Jp\varepsilon}_{\tilde{v}\tilde{j}K,v'j'K'}\right], \tag{10}$$

$$\mathbf{U}^{\pm Jp\varepsilon}_{vjK,v'j'K'}=2\Delta_{v_1j_1,v_2j_2}\Delta_{v'_1j'_1,v'_2j'_2}\left[\mathbf{U}^{Jp\varepsilon}_{vjK,v'j'K'}\pm\varepsilon(-1)^{j_{12}}\mathbf{U}^{Jp\varepsilon}_{\tilde{v}\tilde{j}K,v'j'K'}\right], \tag{11}$$

where the unsymmetrized one can be calculated by[5]

$$\mathbf{V}^{Jp\varepsilon}_{vjK,v'j'K'}=\delta_{KK'}\langle v_1j_1|\langle v_2j_2|\langle j_1j_2j_{12}K|\Delta V|j'_1j'_2j'_{12}K'\rangle|v'_2j'_2\rangle|v'_1j'_1\rangle, \tag{12}$$

$$\begin{aligned}\mathbf{U}^{Jp\varepsilon}_{vjK,v'j'K'}=\delta_{vv'}\delta_{jj'}\Big\{&\delta_{KK'}\left[J(J+1)+j_{12}(j_{12}+1)-2K^2\right]\\&-\delta_{K',K+1}\sqrt{1+\delta_{K,0}}\lambda^+_{JK}\lambda^+_{j_{12}K}-\delta_{K',K-1}\sqrt{1+\delta_{K,1}}\lambda^-_{JK}\lambda^-_{j_{12}K}\Big\},\end{aligned} \tag{13}$$

in which $\lambda^{\pm}_{JK}=\sqrt{J(J+1)-K(K\pm1)}$ and $\lambda^{\pm}_{j_{12}K}=\sqrt{j_{12}(j_{12}+1)-K(K\pm1)}$. The CC equations can be solved effectively by applying the log-derivative method developed by Johnson[6] and Manolopoulos[7], with a set of given J, P_{ex}, p, ε and total energy E. In this method, the log derivative matrix is propagated up to a matching distance and the asymptotic boundary conditions are employed to extract the scattering matrix (S-matrix).

However, since all the K-labeled channels are coupled in the matrix, the computation becomes unaffordable for calculations with large J value. In this work, the coupled-states approximation including only the nearest neighbor Coriolis couplings (CSA-NNCC) approach is thus employed in the dynamical calculations. In this approximation approach, the propagation is

divided into K-labeled processes, in which only the nearest neighbor angular basis functions from $K-1$ to $K+1$ are included. This approach is better than the original coupled states approximation (CSA)[8, 9], in which all Coriolis couplings are discarded. After each propagation, only the S-matrix elements of K-initial state in each K-labeled propagation are used to generate the state-to-state probability.

The transition probability from the initial $(v_1, j_1;\ v_2, j_2)$ to a final one $(v_1', j_1'; v_2', j_2')$ CMSs is given below:

$$P^{J}_{v_1' j_1' v_2' j_2' \leftarrow v_1 j_1 v_2 j_2}(E_c) = \frac{\left(1+\delta_{v_1 v_2}\delta_{j_1 j_2}\right)\left(1+\delta_{v_1' v_2'}\delta_{j_1' j_2'}\right)}{2\left(2j_1+1\right)\left(2j_2+1\right)} \times \sum_{P_{ex} p\varepsilon}\sum_{K}\sum_{K'=K-1}^{K+1}\sum_{j_{12} j_{12}'}\left|S^{\pm Jp\varepsilon(K)}_{v'j'K',vjK}(E_c)\right|^2 . \qquad (14)$$

Finally, we note that the factor of 2 in the denominator of Supplementary Eq. (14) originates from the equal weight of $P_{ex} = \pm 1$ cases, which is different from the algorithm employed in the H_2–H_2 system[10].

The validity of the CSA-NNCC approach for this system was re-examined. We calculated state-to-state probabilities for $J(P_{ex}p\varepsilon)$=10(+−+) at the total energy of E=1.133 eV, which makes the collision energy E_c=0.123 eV for the initial CMS (1, 1; 0, 1), by using the rigorous CC, original CSA[8, 9], and the CSA-NNCC approach[11], respectively. Taking the rigorous CC probabilities as reference, the RMSE from a single initial CMS to all final CMSs is evaluated as,

$$\mathrm{RMSE}_{vj} = \sqrt{\sum_{v'j'} \frac{\left(P^{\mathrm{appox}}_{v'j' \leftarrow vj} - P^{\mathrm{rigorous}}_{v'j' \leftarrow vj}\right)^2}{N^2-1}}, P^{\mathrm{rigorous}}_{v'j' \leftarrow vj} > 0.01, \qquad (15)$$

where N is total number of final CMSs whose probabilities are larger than 0.01, and $P^{\mathrm{rigorous}}_{v'j' \leftarrow vj}$ and $P^{\mathrm{approx}}_{v'j' \leftarrow vj}$ are probabilities of rigorous and approximation methods, respectively. Supplementary

Figure 2 shows RMSEs of all 107 open initial CMSs at the selected energy and parity, and they are arranged by their j_1+j_2 values from high to low. The RMSEs calculated out by CSA-NNCC approach are systematically smaller than those by CSA, which are sometimes large especially when the initial CMSs have high j_1+j_2 values. Since the RMSEs by CSA-NNCC are less than 0.01 in almost all cases, this approach is validated and thus employed in this work.

Supplementary Note 2

The quasi-classical trajectory (QCT) calculations for the HF(v_1=0, j_1=0)+ HF(v_2=0, j_2=0) collision were performed using the VENUS code. The trajectories were initiated with 10 Å separation between two HF molecules, and terminated when they reached a separation of 10 Å again. The propagation time step was selected to be 0.1 fs which conserved the energies better than 0.01 kcal mol^{-1} for all trajectories. The trajectories were halted if the propagation time is exceptionally long (10.0 ps). The maximal impact parameter b_{max} was determined by the maximal total angular momentum $J_{\max}$,

$$b_{\max}^2 \approx J_{\max}(J_{\max} + 1)/k^2 \quad (16)$$

in which $k^2\hbar^2 = 2\mu E_c$. Here, $b_{\max}$ are chosen to be 7.143 Å. In total, 19 316 trajectories have been calculated.

Supplementary Figure 3 displays the lifetime distributions for trajectories with different numbers of inner turning points at E_c=0.05 kcal/mol. There are about 10% of trajectories undergo trapping with more than one inner turning point. These trajectories have significantly longer lifetimes, enabling energy randomization.

Supplementary References

1. Wu, Q., Zhang, D. H. & Zhang, J. Z. H. 6D quantum calculation of energy levels for HF stretching excited $(HF)_2$. *J. Chem. Phys.* **103**, 2548-2554 (1995).

2. Zhang, J. Z. H. *Theory and Application of Quantum Molecular Dynamics*. World Scientific (1999).

3. Zare, R. N. *Angular Momentum*. Wiley (1988).

4. Lin, S. Y. & Guo, H. Full-dimensional quantum wave packet study of rotationally inelastic transitions in H_2 + H_2 collision. *J. Chem. Phys.* **117**, 5183 (2002).

5. Zhang, D. H., Wu, Q., Zhang, J. Z. H., von Dirke, M. & Bačić, Z. Exact full-dimensional bound state calculations for $(HF)_2$, $(DF)_2$, and HFDF. *J. Chem. Phys.* **102**, 2315 (1995).

6. Johnson, B. R. The multichannel log-derivative method for scattering calculations. *J. Comput. Phys.* **13**, 445-449 (1973).

7. Manolopoulos, D. E. An improved log derivative mehtod for inelastic scattering. *J. Chem. Phys.* **85**, 6425-6429 (1986).

8. McGuire, P. & Kouri, D. J. Quantum mechanical close coupling approach to molecular collisions. j_z-conserving coupled states approximation. *J. Chem. Phys.* **60**, 2488-2499 (1974).

9. Pack, R. T. Space-fixed vs body-fixed axes in atom-diatomic molecule scattering. Sudden approximations. *J. Chem. Phys.* **60**, 633-639 (1974).

10. Quéméner, G. & Balakrishnan, N. Quantum calculations of H_2–H_2 collisions: From ultracold to thermal energies. *J. Chem. Phys.* **130**, 114303 (2009).

11. Yang, D., Hu, X., Zhang, D. H. & Xie D. An improved coupled-states approximation including the nearest neighbor Coriolis couplings for diatom-diatom inelastic collision. *J. Chem. Phys.* **148**, 084101 (2018).
